# Patient-Sharing Relations in the Treatment of Diabetes and Their Implications for Health Information Exchange: Claims-Based Analysis

**DOI:** 10.2196/12172

**Published:** 2019-04-12

**Authors:** Georg Duftschmid, Christoph Rinner, Simone Katja Sauter, Gottfried Endel, Peter Klimek, Christoph Mitsch, Harald Heinzl

**Affiliations:** 1 Section for Medical Information Management Center for Medical Statistics, Informatics, and Intelligent Systems Medical University of Vienna Vienna Austria; 2 Main Association of Austrian Social Security Institutions Vienna Austria; 3 Section for Science of Complex Systems Center for Medical Statistics, Informatics, and Intelligent Systems Medical University of Vienna Vienna Austria; 4 Complexity Science Hub Vienna Vienna Austria; 5 Department of Ophthalmology and Optometry Medical University of Vienna Vienna Austria; 6 Section for Clinical Biometrics Center for Medical Statistics, Informatics, and Intelligent Systems Medical University of Vienna Vienna Austria

**Keywords:** health information exchange, professional-patient relations, diabetes mellitus, Austria

## Abstract

**Background:**

Health information exchange (HIE) among care providers who cooperate in the treatment of patients with diabetes mellitus (DM) has been rated as an important aspect of successful care. Patient-sharing relations among care providers permit inferences about corresponding information-sharing relations.

**Objectives:**

This study aimed to obtain information for an effective HIE platform design to be used in DM care by analyzing patient-sharing relations among various types of care providers (ToCPs), such as hospitals, pharmacies, and different outpatient specialists, within a nationwide claims dataset of Austrian DM patients. We focus on 2 parameters derived from patient-sharing networks: (1) the principal HIE partners of the different ToCPs involved in the treatment of DM and (2) the required participation rate of ToCPs in HIE platforms for the purpose of effective communication.

**Methods:**

The claims data of 7.9 million Austrian patients from 2006 to 2007 served as our data source. DM patients were identified by their medication. We established metrics for the quantification of our 2 parameters of interest. The principal HIE partners were derived from the portions of a care provider’s patient-sharing relations with different ToCPs. For the required participation rate of ToCPs in an HIE platform, we determine the concentration of patient-sharing relations among ToCPs. Our corresponding metrics are derived in analogy from existing work for the quantification of the continuity of care.

**Results:**

We identified 324,703 DM patients treated by 12,226 care providers; the latter were members of 16 ToCPs. On the basis of their score for 2 of our parameters, we categorized the ToCPs into *low*, *medium*, and *high*. For the *most important HIE partner* parameter, pharmacies, general practitioners (GPs), and laboratories were the representatives of the top group, that is, our care providers shared the highest numbers of DM patients with these ToCPs. For the *required participation rate of type of care provide (ToCP) in HIE platform* parameter, the concentration of DM patient-sharing relations with a ToCP tended to be inversely related to the ToCPs member count.

**Conclusions:**

We conclude that GPs, pharmacies, and laboratories should be core members of any HIE platform that supports DM care, as they are the most important DM patient-sharing partners. We further conclude that, for implementing HIE with ToCPs who have many members (in Austria, particularly GPs and pharmacies), an HIE solution with high participation rates from these ToCPs (ideally a nationwide HIE platform with obligatory participation of the concerned ToCPs) seems essential. This will raise the probability of HIE being achieved with any care provider of these ToCPs. As chronic diseases are rising because of aging societies, we believe that our quantification of HIE requirements in the treatment of DM can provide valuable insights for many industrial countries.

## Introduction

### Background

Health information exchange (HIE) has been found to improve quality of care in general [1], and it constitutes an important aspect of the successful treatment of diabetes mellitus (DM) in particular [2-6]. Common HIE solutions are disease-specific DM information systems [2,6], as well as general HIE platforms at the regional [7] or national [8] level.

The characteristics of DM patient-sharing relations among care providers can provide useful information for the design and selection of an efficient HIE solution for the treatment of DM. According to Barnett et al, the likelihood of 2 care providers having an information-sharing relation increases with the number of patients they share [9]. Generalizing this finding from individual care providers to *types of care providers* (ToCPs), such as general practitioners (GPs), pharmacies, hospitals, and different types of specialists, we reason that those ToCPs with whom care providers share most of their DM patients should be integral parts of HIE solutions for DM treatment. Furthermore, the concentration of DM patient-sharing relations among different ToCPs allows inferences about the required participation rates of ToCPs in HIE solutions. As we will explain in the section entitled *Measurement of the required participation rates of ToCPs in an HIE solution*, concentration values and required participation rates are inversely related.

Earlier work addressed the associations of patient-sharing relations with health care expenditure, utilization, quality of care [10], interacting drug prescriptions [11], as well as medication costs and patient health status [12]. To our knowledge, patient-sharing networks (PSNs) have not yet been analyzed to gain insights for HIE solutions, except for an earlier study we performed on the use of the Austrian electronic health record system ELGA (acronym for German *Elektronische Gesundheitsakte*) [13].

### Objectives

This study spans across a wider range of aspects than the study by Sauter et al [13]: we now consider all ToCPs who provided health services to our patient cohort rather than considering primary care physicians alone. We also added an analysis of the concentration of patient-sharing relations among different ToCPs, and we suggest a corresponding metric for this purpose. We now focus on DM to the extent that DM care teams depend on HIE [2-6], and thus minimize the inclusion of data concerning random relations among care providers who treat the same patient for unrelated reasons.

We present, for the first time, a comprehensive quantification of the requirements for HIE in the treatment of DM patients, on the basis of a nationwide dataset. The aims of this report were the following:

1. To identify the most important HIE partners of the different ToCPs involved in DM treatment on a large scale by analyzing DM patient-sharing relations among care providers on a nationwide basis in Austria.
2. To identify the required participation rate of ToCPs in HIE solutions to achieve effective communication among care providers in the context of DM management, by analyzing the concentration of DM patient-sharing relations among ToCPs.

These characteristics of DM patient-sharing relations can serve as input for the design of HIE solutions, and they can serve as a decision-making aid for care providers in selecting the most suitable HIE platform when several competing platforms exist in their area. We aim to obtain information concerning the required participants and participation rates of HIE solutions for the treatment of DM. Details of implementation, such as system architecture, interface design, or security mechanisms are not included in the scope of this work.

## Methods

### Data Source

Deidentified claims data of the Main Association of Austrian Social Security Institutions constituted our data source. These included outpatient (GPs, specialists, and pharmacies) as well as inpatient care (see Table 1 for numbers of care providers per type of care provider, ToCP) of 7.9 million persons from all age groups, who were insured by one of the public Social Security Institutions in Austria and had one or more contacts with a care provider between 2006 and 2007. Around 95% of the Austrian population at the time are covered by the database. The small gap results from a few insurance carriers not covered by the database and patients excluded because of inconsistent data for gender or year of birth.

This study was approved by the ethics committee of the Medical University of Vienna (#1903/2017).

All database queries and calculations were implemented using PostgreSQL version 9.4 (PostgreSQL Global Development Group). In total, our SQL script had 858 lines. The calculation of our concentration metrics usual provider cooperation (UPCo) and concentration of cooperation (COCo) is shown in Multimedia Appendix 1.

### Identification of Study Patients

The Austrian health care system does not prescribe the documentation of outpatient diagnoses for reimbursement purposes. Therefore, we identified DM patients on the basis of their medication. In other words, we focused on DM patients undergoing pharmaceutical treatment.

Patients were eligible when at least two diabetes-specific medications had been dispensed to them between 2006 and 2007. In accordance with Chini et al, 9 ATC (Anatomical Therapeutic Chemical Classification System with Defined Daily Doses) codes of the groups *A10A: insulins and analogues* and *A10B: oral antidiabetics* were considered diabetes-specific [14].

Patients below 20 years of age in 2006 were excluded (0.90% (2964/329,313) of our cohort) as, in contrast to older patients, this age group could not be validated well in comparison with a reference population [4]. Patients with missing data about their age (0.49% [1646/329,313] of our cohort) were also excluded.

### Identification of Study Care Providers

We considered all public care providers (those having a contract with a public Austrian Social Security Institution) who provided one or more services to this study’s patients between 2006 and 2007. The ToCP was known in each case. Hospital data were available for inpatient visits but not for walk-in clinics (such as DM ambulances). Dentists were not considered as a substantial part of their services is paid privately by patients, and we lacked the corresponding data.

### Measuring the Need for Health Information Exchange Among Types of Care Providers

Our basic assumption is that the need for HIE with a ToCP is directly related to the number of patients shared with the ToCP. The underlying rationale is that the more patients a care provider shares with other care providers of a particular ToCP, the more external information is generated by this ToCP for the care provider’s patients, and the more important it becomes for the care provider to establish HIE with the respective ToCP.

**Figure 1:**
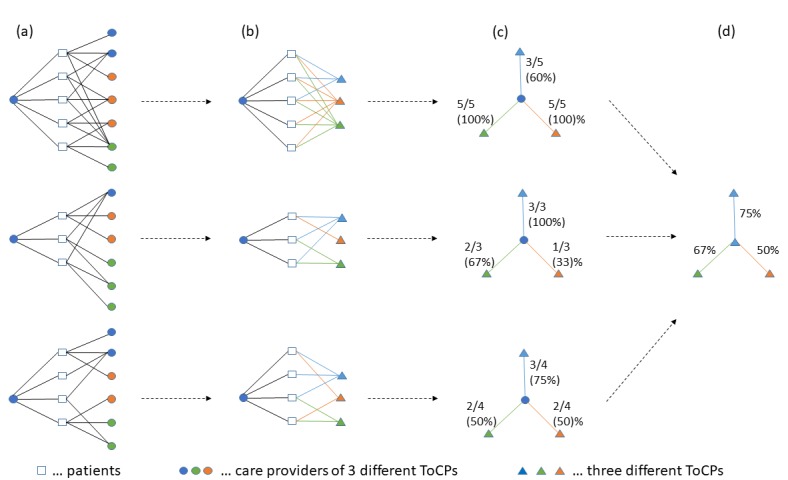
A total of 3 reduced patient-sharing networks (PSNs) are presented. (a) The care providers on the left of each PSN are referred to as the index care providers, whereas the care providers on the right of each PSN are termed linked care providers. An edge between a patient and a linked care provider means that this patient is shared between the index care provider and the linked care provider. Hence, the PSNs show 3 index health care providers of the same ToCP (type of care provider; blue) sharing patients with linked care providers from 3 ToCPs (blue, orange, green). (b) Linked care providers are aggregated per ToCP (shown as colored triangles); an edge between a patient and a linked ToCP means that this patient is shared among the index care provider and at least one linked care provider of this ToCP. Patient-sharing relations are colored according to the linked care providers’ ToCPs. (c) Shared patients per linked ToCP are calculated; edges depict the proportions (percentages) of patients shared by the index care provider with each linked ToCP. (d) Index care providers are aggregated per ToCP (here only blue is available); edges depict the typical (median) percentages of patients shared with linked ToCPs (here blue, orange, and green).

We originate from the PSNs [15] of each care provider. Compared with the study by Landon et al [15], the PSNs are *reduced* to the patient-sharing relations between the observed *index* care provider and other *linked* care providers, as the patient-sharing relations among the linked care providers are not relevant for our metrics. Figure 1 explains how we derived patient-sharing portions among ToCPs from the PSNs.

### Measuring the Required Participation Rates of Types of Care Providers in a Health Information Exchange Solution

We see the concentration of DM patient-sharing relations among ToCPs as an indicator that is inversely related to the ToCPs’ required participation rates in an HIE solution. If, for example, each DM patient of a care provider gets her medication at a different pharmacy, a broad participation of pharmacies in the HIE will be necessary to allow the exchange of medication data with practically any pharmacy that a DM patient might visit. However, if a care provider’s DM patients are referred to a small number of laboratories, the care provider might get by with an HIE solution with restricted participation of laboratories as long as it covers the laboratories typically visited by her DM patients.

In this study, 2 care providers who share 1 or more patients are referred to as *cooperating* care providers. For measuring the COCo among care providers, we were inspired by the existing metrics for the quantification of the *continuity of care* [16,17]. From the *usual provider continuity (UPC)* [17] that is the most frequently applied continuity of care (COC) index in literature [18], we derived our new metric *UPCo* by exchanging UPC’s patient contacts with patient-sharing relations. The new metric is then defined in the following manner:

UPCo = n
_i_ / N

n_i_ is the number of patients the index care provider shares with the *usual linked care provider*, and N is the total number of patient-sharing relations between the index care provider and all linked care providers in a specific time period. We determine a usual linked care provider for each ToCP and define the usual linked care provider for ToCP X (following a typical UPC procedure) as the linked care provider of ToCP X with whom the index care provider shares the highest number of her patients. If 2 or more linked care providers share the same maximum number of patients with the index care provider, 1 of them is arbitrarily chosen as the usual linked care provider, whereas the others are treated as regular linked care providers. The UPCo for ToCP X then reflects the percentage of an index care provider’s patient-sharing relations within ToCP X that are associated with the usual linked care provider of ToCP X.

**Figure 2:**
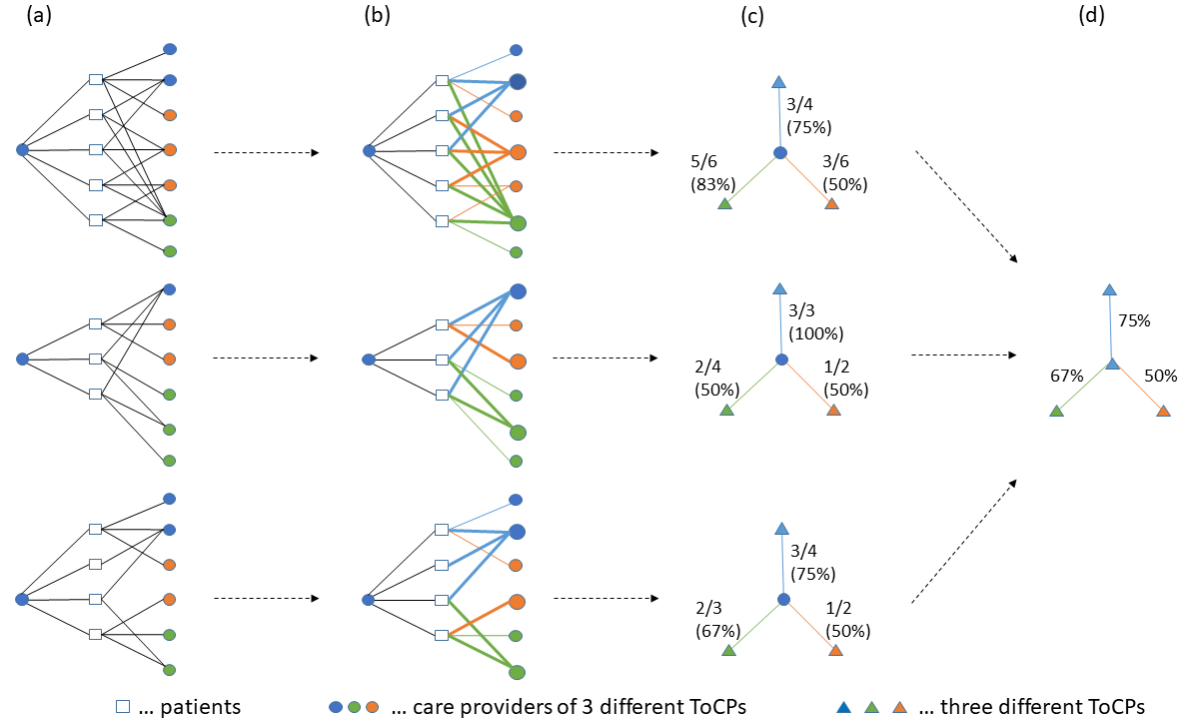
Calculation of typical usual provider cooperation (UPCo) values. (a) We originate from the same patient-sharing network as shown in Figure 1 a. (b) Index care providers’ usual linked care providers (shown as enlarged dots) are determined separately for each ToCP (type of care provider), thereby ties are broken randomly. Patient-sharing relations are colored according to the linked care providers’ ToCPs; relations with the usual linked care provider are emphasized additionally. (c) UPCo values (depicted on edges both as proportions and percentages) are calculated for each ToCP. By way of an example, the UPCo value of 75% in the topmost network indicates that 3 out of 4 patient-sharing relations between the first index care provider and the linked blue care providers are shared with the usual linked blue care provider. (d) Index care providers are aggregated per ToCP; edges depict the typical (median) UPCo values with linked ToCPs. Trivial UPCo values of 100% in case of an index care provider who shared only 1 single patient with only 1 single-linked care provider within a particular ToCP were not considered in our analysis. This was the case in 5.7% of the patient-sharing relations between index care providers and linked ToCPs.

Figure 2 explains how we derived typical UPCo values among ToCPs from the PSNs.

As a crosscheck of the robustness of our UPCo measurements, we additionally calculated a second metric derived from the frequently used COC [16]. The corresponding results were very similar to the UPCo measurements (see Multimedia Appendix 2 for details).

## Results

### Identified Study Patients

We identified 324,703 DM patients (3.92% (324,703/8,280,711) of the mean Austrian population between 2006 and 2007) who satisfied our inclusion and exclusion criteria (see section *Identification of study patients*).

### Identified Study Care Providers

We identified 12,226 care providers who satisfied our inclusion and exclusion criteria (see section entitled *Identification of study care providers*). Table 1 shows the distribution of these providers across the different ToCPs.

### Need for Health Information Exchange Among Types of Care Providers

**Figure 3:**
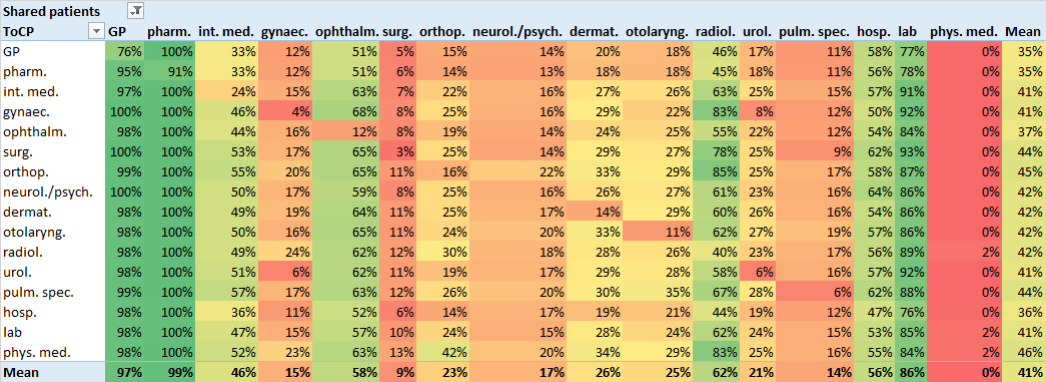
Typical (median) portion of patients shared by an index care provider of the ToCP shown in the leftmost column, with linked care providers of the ToCP shown in the topmost row. ToCPs are sorted by the number of care providers per ToCP (compare Table 1). Cells are color-coded with colors ranging from dark green for high values to dark red for low values. Intermediate values are shown in graded color intensities. The bottom row and the right-most column show the mean value of the corresponding column’s respectively row’s values. As a measure of variance, interquartile ranges are given in Multimedia Appendix 3. GP: general practitioner; pharm: pharmacy; int med: internal medicine; gynaec: gynecology; ophthalm: ophthalmology; surg: surgery; orthop: orthopedics; neurol/psych: neurology/psychiatry; dermat: dermatology; otolaryng: otolaryngology; radiol: radiology; urol: urology; pulm: pulmology; hosp: hospital; lab: laboratory; phys med: physical medicine.

Figure 3 shows the results of the procedure described in Figure 1, that is, the median portions of DM patients shared by care providers of any 2 ToCPs.

The rows of Figure 3 show median portions of patients *shared by an index care provider of a particular ToCP*. As an example, the row entitled *GP* shows how many of her patients a GP typically shares with linked care providers of each ToCP. The columns of Figure 3 show median portions of patients *shared with a linked care provider of a particular ToCP*. For instance, the column entitled *GP* shows how many of their patients the index care providers of each ToCP typically share with a GP.

The bottom right cell of Figure 3 shows that the average median percentage of DM patients shared by our index care providers with any single-linked ToCP was 41%. According to the right-most column entitled *Mean*, the corresponding range was between 35% (GPs, pharmacies) and 46% (physical medicine).

According to the bottom row entitled *Mean*, linked ToCPs differed strongly in the average median percentages of patients that index care providers shared with them. We grouped the linked ToCPs according to these portions and assigned each of them to 1 of the 3 categories: (0%-33%), (33%-66%), and (66%-99%), on the basis of the observed range. Pharmacies (99%), GPs (97%), and laboratories (86%) were located in the top category. Radiologists (62%), ophthalmologists (58%), hospitals (56%), and internal medicine specialists (46%) were assigned to the middle category. All other linked ToCPs belonged to the bottom category.

We noted similar values within each column of Figure 3, except for the main diagonal. This means that the number of patients shared with any single-linked ToCP was similar for all index ToCPs. In most cases, cells along the main diagonal of Figure 3 contain low values compared with the other values in their column. In other words, care providers usually share more of their patients with care providers of other ToCPs than with their own ToCP.

### Required Participation Rates of Types of Care Providers

**Figure 4:**
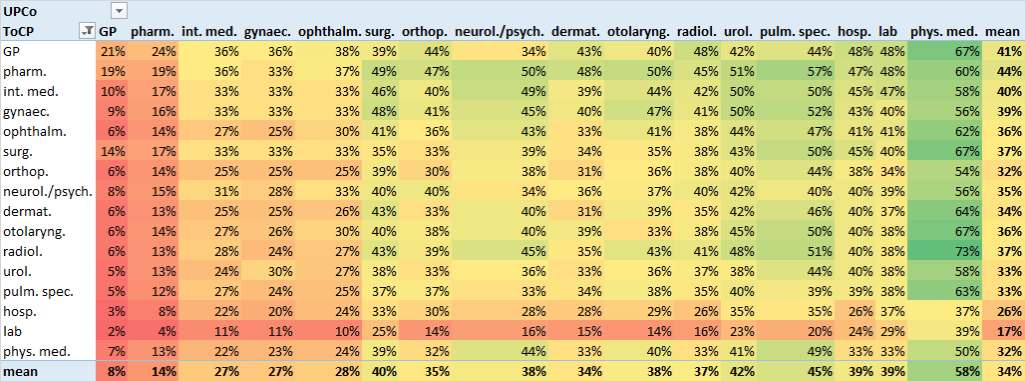
Typical (median) UPCo values of an index care provider of the ToCP shown in the left-most column with a linked care provider of the ToCP shown in the topmost row. ToCPs are sorted by the number of care providers per ToCP (compare Table 1). Cells are color-coded with colors ranging from dark green for high values to dark red for low values. Intermediate values are shown in graded color intensities. The bottom row and the right-most column show the mean value of the corresponding column’s respectively row’s values. As a measure of variance, interquartile ranges are given in Multimedia Appendix 3. GP: general practitioner; pharm: pharmacy; int med: internal medicine; gynaec: gynecology; ophthalm: ophthalmology; surg: surgery; orthop: orthopedics; neurol/psych: neurology/psychiatry; dermat: dermatology; otolaryng: otolaryngology; radiol: radiology; urol: urology; pulm: pulmology; hosp: hospital; lab: laboratory; phys med: physical medicine.

Figure 4 shows the results of the procedure described in Figure 2, that is, the median UPCo values among care providers of any 2 ToCPs.

The bottom right cell of Figure 4 shows that the average median concentration of our index care providers’ shared patients on the usual linked care provider of any single-linked ToCP was 34%. The strongest deviations (compare right-most column *mean*) from this average occurred for laboratories (17%) and pharmacies (44%).

**Table 1:** Numbers of study care providers per type of care provider.

Type of care provider	n
General Practitioner	4892
Pharmacy	2240
Internal medicine	949
Gynecology	778
Ophthalmology	510
Surgery	441
Orthopedics	407
Neurology/psychiatry	391
Dermatology	329
Otolaryngology	299
Radiology	277
Urology	258
Pulmology	165
Hospital	132
Laboratory	102
Physical medicine	56

The bottom row entitled *mean* shows the range of average median UPCo values with the different linked ToCPs. We grouped the linked ToCPs according to these values and assigned them to 1 of the 3 categories: (8%-25%), (25%-42%), and (42%-58%), on the basis of the observed range.

Physical medicine (58%), pulmology (45%), and urology (42%) were located in the top category. GPs (8%) and pharmacies (14%) were the only ToCPs in the bottom category. All other ToCPs were assigned to the middle category.

Overall, Figure 4 shows a rather smooth increase of UPCo values from left to right. This seems to indicate that the concentration of patient-sharing relations is associated with the number of care providers per linked ToCP. The row entitled *lab* shows unusual values in this respect; they indicate that the laboratories’ concentration of patient-sharing relations with other ToCPs is rather constantly low, regardless of the number of care providers per linked ToCP.

## Discussion

### Material and Methods

We relied on an established research database as our main data source. The database has been successfully used in various earlier research projects related to HIE [4,13,19]. This study’s population of DM patients was shown to be plausible, on the basis of a comparison with the Austrian Health Survey of 2006/07 [4].

The UPCo and COCo (see Multimedia Appendix 2) metrics used to measure the concentration of patient-sharing relations were simple to calculate and could thus be easily implemented in PostgreSQL. Selecting the linked care provider with whom the index care provider shared the highest number of her patients as the *usual provider* in the calculation of the UPCo seems reasonable to us when aiming for a measure for the concentration of patient-sharing relations.

Barnett and coworkers showed that the likelihood of a professional relationship between 2 care providers increases with the number of patients shared between them [9]. According to the authors, 9 or more shared patients indicate an actual professional relationship with a likelihood of more than 80%. We did not apply a minimum number of shared patients to consider a relationship between 2 care providers as our focus was on HIE with ToCPs. If, for example, a care provider shares each of her patients with a different pharmacy, pharmacies will still be an important HIE partner in our context, and it will be important to know that the COCo with pharmacies is low for this care provider.

We did not perform a stratified analysis for different patient attributes such as age and gender, as it would not provide useful conclusions for our research question. Each care provider typically treats a variety of patients with different characteristics and should be covered by a single HIE solution that is most suitable for all her patients. For HIE solutions that are optimized for different patient groups, it would not be realistic for a care provider to use multiple HIE solutions in parallel.

### Need for Health Information Exchange Among Types of Care Providers

Ideally, each care provider involved in the treatment of DM patients would have access to an HIE platform that would allow her to exchange any health information with any care provider of any type. However, in current practical settings, this is usually not the case. As current platforms typically provide only partial HIE (ie, the content of selected ToCPs is shared or a subset of care providers participates in the HIE platform), it seems reasonable to categorize ToCPs according to their relevance for HIE. This knowledge could help to determine priorities for integrating ToCPs in an HIE platform.

According to Figure 3, we interpret pharmacies, GPs, and laboratories as HIE partners of high priority for any care provider involved in DM treatment insofar as the highest portions of patients are shared with them (compare the columns *GP*, *lab*, and *pharm.* in Figure 3). It should be noted that patient-sharing portions with laboratories are highly variable (compare interquartile ranges for the column entitled *lab* in Multimedia Appendix 3). In other words, laboratories might not be top priority HIE partners for all care providers.

Radiologists, ophthalmologists, hospitals, and internal medicine specialists might be classified as HIE partners of middle-rate priority in DM treatment. Variations are rather high among radiologists and internal medicine specialists (compare interquartile ranges for the corresponding columns in Multimedia Appendix 3), which means that they might be less important HIE partners for some care providers. Rather, a few DM patients are shared with all other ToCPs compared with the ToCPs rated as high or middle-rate priority (see above); thus, HIE will be needed less frequently here.

For an exemplary application of this study’s results, we analyzed the recently introduced Austrian electronic health record (EHR) system ELGA [20] for its suitability in the treatment of DM. ELGA currently supports the exchange of (1) medication data prescribed by any ToCP and dispensed by pharmacies, (2) lab reports generated by hospital-based and outpatient laboratories, (3) radiology reports generated by hospitals and outpatient radiologists, and (4) hospital discharge letters. In other words, ELGA covers all ToCPs whom we rated as high-priority or medium-priority HIE partners, although GPs, ophthalmologists, and internal medicine specialists currently might only feed medication data into ELGA.

The fact that care providers typically share more of their patients with care providers of other types than with their own type (compare main diagonal of Figure 3) might primarily be explained by the fact that the payment systems of most Austrian social health insurances limit the access to care providers per ToCP (only 1 care provider per ToCP might be accessed during 1 accounting period).

### Required Participation Rates of Types of Care Providers

In view of the postulated inverse relation of UPCo values with required participation, GPs and pharmacies would require high participation rates in the HIE platform. High participation rates might also be recommended for members of the middle UPCo category (int. med., gynec., ophthalm., dermat., orthop., radiol., neurol./psych., otolaryng., hosp., lab., and surg.). These desired participation rates could be reliably achieved by means of a national HIE platform with obligatory participation of the aforementioned ToCPs. In Austria, ELGA ensures this condition through obligatory participation of all public care providers.

The apparent association of the concentration of patient-sharing relations with the number of care providers per linked ToCP seems intuitive. For instance, if 1 of our index care providers shared DM patients with a linked ToCP of *physical medicine*, the patients could choose among only 56 physical medicine specialists. If we assume that care providers of a ToCP are distributed rather evenly in geographic terms (public care providers should reasonably be distributed in a way that allows them to be accessed homogeneously by the entire population), the 56 physical medicine specialists will usually be located farther apart from each other than the 4892 GPs. It would thus be obvious that many of the index care providers’ patients concentrated on the physical medicine specialist who is located closest to the index care provider. This would explain the high UPCo values in the column entitled *phys. med.* in Figure 4. In contrast, DM patients shared with GPs would naturally be divided among several different GPs located in close vicinity to the index care provider. This would explain the low UPCo values in the column entitled *GP* in Figure 4.

Another explanation for the characteristics of Figure 4 could be the *patient sending/receiving role* of the ToCPs. For instance, GPs and internal medicine specialists have a *patient sending role*. They act as *gatekeepers* and are the first to be visited by a DM patient in a treatment chain. When the patients of a GP have to visit other care providers in due course, similar patterns in selecting these care providers (and thus high UPCo values) could result from recommendations of the GP. In contrast, a care provider with a *patient receiving role* (such as laboratories and pharmacies) has less influence on whose patients are sent to her, resulting in low UPCo values. This explanation would be in accordance with the high UPCo values in the rows entitled *GP* and *int. med.* in Figure 4, and it would be in accordance with the low UPCo values in the row entitled *lab*. However, it would be in conflict with the high UPCo values in the row entitled *pharm.*.

### Related Work

In the context of diabetes-specific HIE, several authors concentrated on the patients’ role in information sharing [21,22]. HIE between DM patients and care providers was examined with a focus on sharing medication data [23], email communication [24], and patient preferences [25].

Koopman and coworkers name a set of data elements that are relevant for outpatient family physicians and general internal medicine physicians in the treatment of DM patients [26]. However, they neither address how these data elements were identified nor address which ToCPs should deliver the corresponding values.

Huebner-Bloder and coworkers identified 446 relevant data elements in the treatment of DM and grouped these in 9 categories [27]. They used a triangulation design that was mainly based on documentation analysis in 3 DM outpatient clinics and interviews with 6 internists specialized in DM. The identified data elements originate from GPs, internal medicine physicians, ophthalmologists, nephrologists, neurologists, gynecologists, psychiatrists, dermatologists, hospitals, laboratories, and from the patient’s self-monitoring. The ToCPs identified by them as being relevant in the treatment of DM thus constitute a subset of our ToCPs, except for nephrologists (who are a part of the ToCP *internal medicine* in our claims data) and patient-reported data (not considered in our claims data).

According to Joshy and Simmons, HIE between systems of GPs and hospitals are crucial factors for the success of DM information systems [2]. They further state that “pharmacy data, lab measurements, retinal screening, and home blood glucose monitoring data are increasingly being linked into diabetes information systems.” This fits with this study’s results insofar as we identified GPs, pharmacies, and laboratories as high-priority HIE partners in the treatment of DM, as well as hospitals and ophthalmologists as middle-priority HIE partners. Patient-reported data were not considered in this study.

Existing HIE platforms only partly cover the information needs of care providers. According to a recent study, only 58% of the analyzed DM information systems provided HIE with hospitals, 22% provided HIE with primary care, and only 3% provided HIE with hospitals and primary care [28]. In their review of regional HIE platforms, Mäenpää et al conclude that the latter provide inadequate access to patient-relevant clinical data [29]. Nationwide EHR systems, which are operated as national HIE platforms in 59% of the European World Health Organization member states [30], are typically restricted to the exchange of patient summaries or selected document types [8].

### Limitations

One of our basic assumptions was that those ToCPs with whom care providers share most of their DM patients should be considered high-priority HIE partners. Even though this assumption seems intuitive, in the individual case, there might also be care providers from ToCPs with low patient-sharing portions, who possess patient information of high importance. Furthermore, the need for HIE might differ among certain combinations of ToCPs and thus not be naturally reflected by patient-sharing rates. As an example, laboratories typically share 98% of their DM patients with GPs, whereas GPs *only* share 77% of their patients with laboratories (compare Figure 3). Nevertheless, it might be more important for the GP to receive a lab result (and then add it to the patient’s local EHR) than for the laboratory to electronically receive the request for a particular test (this could probably also be solved conventionally without serious detriment).

Hospital visits could only be considered from the inpatient domain in our analysis; data from hospital outpatient departments were not included in our data source. As hospital outpatient departments are frequently visited by Austrian DM patients, hospitals are probably underrepresented as ToCPs in this study. Furthermore, visits to private care providers were not taken into account as the corresponding data were incomplete in our data source. This might have led to an underrepresentation of those ToCPs with large numbers of private care providers in Austria, such as physical medicine, surgery, and neurology/psychiatry.

We only focused on DM patients; therefore, our insights concerning HIE can only be applied to the treatment of DM. However, as a next step, we intend to repeat the analysis for other chronic diseases to see whether there are general *patterns* related to the design and selection of HIE platforms in the treatment of chronically ill patients.

### Conclusions

The results of this study provide insights for 2 different types of actors in the HIE of DM patients.

First, implementers and providers of HIE platforms who strive to support DM treatment should ensure that they integrate GPs, pharmacies, and laboratories from the start, as these constitute HIE partners of high priority for all ToCPs. Radiologists, ophthalmologists, hospitals, and internal medicine specialists should be integrated into the HIE platforms in the second step. The remaining ToCPs seem to be HIE partners of lower priority in the treatment of DM and could thus be integrated in the final step, if resources permit. Furthermore, this study’s results seem to suggest that DM patients shared with ToCPs who have many members (in our case, particularly GPs and pharmacies) are divided among many different care providers of these ToCPs. We conclude that, for implementing HIE with ToCPs who have many members, it would be essential to have an HIE solution with high participation rates of these ToCPs (ideally a nationwide HIE platform with obligatory participation of the concerned ToCPs). This would increase the chances of HIE with any cooperating care provider of these ToCPs.

In Austria, ELGA satisfies these demands and thus serves as a suitable HIE platform for DM treatment. It could become even more useful if, besides medication data, further information registered by GPs, ophthalmologists, and internal medicine specialists was provided.

Second, care providers using a DM-specific HIE platform might gain insights from this study’s results for *their* index ToCP (ie, *their* row of Figure 3 and Figure 4). This study’s results provide information about a care provider’s general DM-related HIE characteristics with respect to all linked ToCPs. For instance, radiologists seem to be important HIE partners for orthopedists as they typically share 85% of their DM patients with radiologists, according to the row entitled *orthop.* in Figure 3. However, for hospitals, radiologists appear to be HIE partners of rather low priority (only 44% shared DM patients). Furthermore, the rather constantly low UPCo values of laboratories indicate that a laboratory’s shared DM patients are usually divided among several care providers for each linked ToCP. This suggests that, in the context of DM treatment in Austria, laboratories benefit from being connected to an HIE solution with high participation rates of other ToCPs. Being able to transmit test results to practically any requesting care provider via the HIE solution will widen the laboratory’s catchment area of cooperating care providers and thus be commercially useful.

## Appendix

Multimedia Appendix 1 PostgreSQL script for the calculation of the metrics usual provider cooperation and concentration of cooperation.

Multimedia Appendix 2 The concentration of cooperation metric.

Multimedia Appendix 3 Interquartile ranges for portions of shared patients and usual provider cooperation values.

## Abbreviations

COC: continuity of care index
COCo: concentration of cooperation
DM: diabetes mellitus
EHR: electronic health record
ELGA: Elektronische Gesundheitsakte (electronic health record)
GP: general practitioner
HIE: health information exchange
PSN: patient-sharing network
ToCP: type of care provider
UPC: usual provider continuity
UPCo: usual provider cooperation
