# Supplementary material for: Patient-Sharing Relations in the Treatment of Diabetes and Their Implications for Health Information Exchange: Claims-Based Analysis
Source: JMIR Med Inform. 2019 Apr 12;7(2):e12172. doi: 10.2196/12172 (PMC6484263; doi:10.2196/12172)
Supplement: Multimedia Appendix 2 [file medinform_v7i2e12172_app2.pdf]

Multimedia Appendix 2

The Concentration of Cooperation (COCo) metric

We additionally calculated a second metric for the concentration of cooperation that we derived from the "Continuity of Care Index (COC)" [20] by exchanging COC's patient contacts with patient-sharing relations. The new metric is then defined as

$$\mathrm{COCo} = \frac{\sum_{i=1}^{k} n_i^2 - N}{N\,(N-1)}$$

where n_i is the number of patients shared between the index care provider and linked care provider *i*, *k* is the total number of linked care providers with whom the index care provider shares patients, and N is the total number of patient-sharing relations between the index care provider and any other linked care provider in a defined time period.

The COCo yields values between 0 and 1. A value of 1 indicates a high concentration of patient-sharing relations (all patients are shared with one single provider), whereas a value of 0 indicates a low concentration (each patient is shared with a different provider).

We then calculated the networks described in figure 2, this time holding the COCo instead of the UPCo values.

Table 1 depicts the median COCo values between care providers of any two ToCPs.

the results of this procedure.

Table 1. Typical (median) COCo values of an index care provider of the ToCP shown in the leftmost column with a linked care provider of the ToCP shown in the topmost row. ToCPs are sorted by the number of care providers per ToCP (compare table 1). Cells are color-coded with colors ranging from dark green for high values to dark red for low values. Values in between are depicted with gradual color intensities. The bottom row and the right-most column show the mean value of the corresponding column's/row's values.

COCo																	
ToCP	GP	pharm.	int. med.	gynaec.	ophthalm.	surg.	orthop.	neurol./psych.	dermat.	otolaryng.	radiol.	urol.	pulm. spec.	hosp.	lab	phys. med.	mean
GP	0,08	0,09	0,18	0,15	0,21	0,13	0,23	0,14	0,21	0,19	0,30	0,20	0,22	0,28	0,27	0,47	**0,21**
pharm.	0,09	0,08	0,20	0,16	0,22	0,24	0,26	0,28	0,28	0,33	0,29	0,33	0,36	0,28	0,30	0,38	**0,26**
int. med.	0,02	0,04	0,11	0,10	0,12	0,20	0,17	0,22	0,16	0,20	0,21	0,26	0,30	0,22	0,24	0,34	**0,18**
gynaec.	0,01	0,04	0,11	0,04	0,13	0,18	0,17	0,20	0,17	0,24	0,21	0,25	0,32	0,21	0,21	0,33	**0,18**
ophthalm.	0,02	0,04	0,12	0,11	0,12	0,21	0,19	0,23	0,19	0,24	0,21	0,27	0,28	0,22	0,23	0,42	**0,19**
surg.	-	0,01	0,07	0,04	0,06	0,08	0,07	0,11	0,12	0,11	0,13	0,17	0,17	0,19	0,14	0,40	**0,12**
orthop.	0,01	0,03	0,09	0,07	0,09	0,16	0,08	0,17	0,13	0,16	0,18	0,19	0,23	0,18	0,17	0,33	**0,14**
neurol./psych.	0,01	0,03	0,11	0,09	0,12	0,16	0,16	0,09	0,15	0,16	0,19	0,19	0,20	0,20	0,20	0,36	**0,15**
dermat.	0,02	0,04	0,10	0,09	0,12	0,21	0,17	0,21	0,12	0,21	0,19	0,24	0,26	0,21	0,19	0,43	**0,18**
otolaryng.	0,02	0,04	0,12	0,11	0,14	0,20	0,20	0,20	0,19	0,14	0,22	0,24	0,29	0,22	0,22	0,46	**0,19**
radiol.	0,02	0,05	0,16	0,12	0,14	0,24	0,23	0,28	0,22	0,28	0,24	0,32	0,36	0,23	0,22	0,57	**0,23**
urol.	0,01	0,03	0,09	0,10	0,11	0,18	0,16	0,17	0,15	0,16	0,20	0,13	0,24	0,21	0,21	0,35	**0,16**
pulm. spec.	0,02	0,04	0,13	0,10	0,12	0,18	0,20	0,16	0,17	0,21	0,22	0,23	0,17	0,21	0,21	0,41	**0,17**
hosp.	0,01	0,03	0,09	0,08	0,10	0,14	0,13	0,16	0,13	0,15	0,13	0,17	0,19	0,13	0,21	0,11	**0,12**
lab	-	0,01	0,04	0,04	0,04	0,10	0,05	0,06	0,06	0,05	0,07	0,10	0,09	0,10	0,15	0,15	**0,07**
phys. med.	0,02	0,04	0,08	0,09	0,11	0,18	0,13	0,24	0,19	0,20	0,19	0,21	0,30	0,13	0,14	0,27	**0,16**
**mean**	**0,02**	**0,04**	**0,11**	**0,09**	**0,12**	**0,17**	**0,16**	**0,18**	**0,17**	**0,19**	**0,20**	**0,22**	**0,25**	**0,20**	**0,21**	**0,36**	**0,17**

The COCo results are similar to the UPCo results and thus support the insights gained from these measurements of the concentration of cooperation.
