# Supplementary material for: Patient-Sharing Relations in the Treatment of Diabetes and Their Implications for Health Information Exchange: Claims-Based Analysis
Source: JMIR Med Inform. 2019 Apr 12;7(2):e12172. doi: 10.2196/12172 (PMC6484263; doi:10.2196/12172)
Supplement: Multimedia Appendix 3 [file medinform_v7i2e12172_app3.pdf]

Multimedia Appendix 3

Interquartile ranges for portions of shared patients and UPCo values

Table 1 and Table 2 show the interquartile ranges in correspondence to the median values shown in sections “need for HIE between ToCPs” and “required participation rates of ToCPs” respectively.

Table 1. Interquartile ranges for the portions of shared patients. These measures of dispersion amend the median values reported in section “need for HIE between ToCPs”.

Shared patients (IQR)																	
ToCP	GP	pharm.	int. med.	gynaec.	opthalm.	surg.	orthop.	neurol./psych.	dermat.	otolaryng.	radiol.	urol.	pulm. spec.	hosp.	lab	phys. med.	Mean
GP	0,23	0,00	0,18	0,07	0,12	0,08	0,14	0,09	0,13	0,10	0,21	0,09	0,08	0,13	0,36	0,05	0,13
pharm.	0,25	0,13	0,19	0,06	0,10	0,08	0,10	0,07	0,12	0,10	0,20	0,08	0,08	0,10	0,42	0,05	0,13
int. med.	0,08	0,00	0,34	0,14	0,18	0,14	0,25	0,17	0,20	0,17	0,27	0,19	0,22	0,17	0,48	0,06	0,19
gynaec.	0,02	0,00	0,23	0,13	0,17	0,16	0,27	0,18	0,22	0,20	0,23	0,13	0,19	0,16	0,29	0,07	0,17
opthalm.	0,03	0,00	0,17	0,07	0,11	0,10	0,18	0,07	0,14	0,10	0,21	0,10	0,09	0,12	0,26	0,07	0,11
surg.	0,02	0,00	0,40	0,26	0,38	0,15	0,45	0,23	0,44	0,38	0,44	0,38	0,23	0,50	0,40	0,04	0,29
orthop.	0,03	0,00	0,17	0,12	0,14	0,16	0,19	0,14	0,18	0,13	0,16	0,14	0,15	0,18	0,26	0,12	0,14
neurol./psych.	0,02	0,00	0,27	0,15	0,16	0,15	0,25	0,23	0,18	0,16	0,24	0,17	0,16	0,19	0,27	0,05	0,17
dermat.	0,03	0,00	0,16	0,08	0,10	0,11	0,20	0,08	0,13	0,10	0,19	0,11	0,09	0,11	0,20	0,09	0,11
otolaryng.	0,04	0,00	0,16	0,08	0,10	0,11	0,22	0,10	0,15	0,10	0,20	0,10	0,11	0,13	0,13	0,09	0,11
radiol.	0,02	0,00	0,16	0,07	0,09	0,10	0,21	0,07	0,11	0,08	0,33	0,08	0,08	0,10	0,17	0,12	0,11
urol.	0,03	0,00	0,17	0,05	0,12	0,13	0,21	0,08	0,16	0,10	0,24	0,07	0,10	0,14	0,23	0,07	0,12
pulm. spec.	0,02	0,00	0,14	0,07	0,10	0,11	0,19	0,07	0,12	0,09	0,19	0,08	0,07	0,12	0,12	0,09	0,10
hosp.	0,02	0,00	0,17	0,04	0,08	0,06	0,07	0,06	0,06	0,06	0,17	0,07	0,06	0,18	0,13	0,07	0,08
lab	0,02	0,00	0,11	0,26	0,07	0,08	0,19	0,03	0,13	0,08	0,22	0,11	0,06	0,09	0,36	0,08	0,12
phys. med.	0,03	0,00	0,16	0,10	0,15	0,09	0,27	0,11	0,15	0,13	0,13	0,11	0,11	0,17	0,86	0,04	0,16
**Mean**	**0,06**	**0,01**	**0,20**	**0,11**	**0,14**	**0,11**	**0,21**	**0,11**	**0,16**	**0,13**	**0,23**	**0,13**	**0,12**	**0,16**	**0,31**	**0,07**	**0,14**

Table 2. Interquartile ranges for the UPCo values. These measures of dispersion amend the median values reported in section “required participation rates of ToCPs”.

UPCo (IQR)																	
ToCP	GP	pharm.	int. med.	gynaec.	ophthalm.	surg.	orthop.	neurol./psych.	dermat.	otolaryng.	radiol.	urol.	pulm. spec.	hosp.	lab	phys. med.	mean
GP	0,28	0,16	0,24	0,25	0,26	0,31	0,34	0,35	0,30	0,30	0,31	0,30	0,40	0,27	0,31	0,36	0,30
pharm.	0,23	0,15	0,27	0,30	0,27	0,36	0,38	0,35	0,38	0,38	0,33	0,36	0,42	0,25	0,35	0,35	0,32
int. med.	0,15	0,15	0,30	0,31	0,31	0,33	0,40	0,35	0,35	0,37	0,35	0,37	0,45	0,24	0,32	0,43	0,32
gynaec.	0,11	0,16	0,29	0,25	0,30	0,30	0,38	0,32	0,33	0,40	0,30	0,40	0,45	0,25	0,22	0,44	0,31
ophthalm.	0,06	0,10	0,25	0,25	0,29	0,32	0,36	0,34	0,35	0,34	0,31	0,36	0,42	0,23	0,26	0,38	0,29
surg.	0,26	0,16	0,30	0,31	0,31	0,25	0,25	0,25	0,25	0,27	0,25	0,28	0,30	0,25	0,25	0,47	0,28
orthop.	0,09	0,12	0,24	0,23	0,25	0,24	0,27	0,34	0,32	0,28	0,25	0,30	0,38	0,24	0,28	0,41	0,26
neurol./psych.	0,10	0,11	0,24	0,28	0,30	0,27	0,35	0,32	0,34	0,26	0,29	0,32	0,38	0,22	0,26	0,37	0,27
dermat.	0,05	0,10	0,26	0,22	0,24	0,29	0,37	0,30	0,24	0,34	0,26	0,31	0,38	0,26	0,25	0,35	0,26
otolaryng.	0,05	0,11	0,25	0,25	0,28	0,28	0,40	0,32	0,35	0,27	0,31	0,36	0,41	0,24	0,26	0,37	0,28
radiol.	0,04	0,09	0,26	0,24	0,25	0,30	0,39	0,36	0,35	0,35	0,34	0,35	0,43	0,25	0,27	0,37	0,29
urol.	0,06	0,11	0,26	0,24	0,26	0,26	0,39	0,32	0,35	0,30	0,28	0,27	0,39	0,23	0,25	0,36	0,27
pulm. spec.	0,04	0,09	0,24	0,25	0,24	0,23	0,37	0,30	0,37	0,29	0,29	0,30	0,31	0,23	0,24	0,39	0,26
hosp.	0,03	0,08	0,24	0,26	0,27	0,30	0,35	0,35	0,38	0,35	0,30	0,38	0,46	0,18	0,26	0,30	0,28
lab	0,03	0,04	0,08	0,10	0,10	0,22	0,13	0,15	0,12	0,13	0,14	0,17	0,17	0,19	0,23	0,30	0,14
phys. med.	0,05	0,11	0,23	0,19	0,23	0,40	0,34	0,32	0,28	0,23	0,22	0,39	0,47	0,29	0,29	0,33	0,27
**mean**	**0,10**	**0,12**	**0,25**	**0,24**	**0,26**	**0,29**	**0,34**	**0,31**	**0,32**	**0,30**	**0,28**	**0,33**	**0,39**	**0,24**	**0,27**	**0,37**	**0,28**
